# Narrow-band asymmetric THz absorbers and polarizers based on impedance-matched, cloaked quasi-BICs in doped Ge microdisk metasurfaces

**DOI:** 10.1016/j.isci.2026.116169

**Published:** 2026-06-05

**Authors:** Lucía Hidalgo-Arteaga, Jose L. Pura, Braulio García-Cámara, Ángela Barreda, José A. Sánchez-Gil

**Affiliations:** 1 Instituto de Estructura de la Materia (IEM), Consejo Superior de Investigaciones Científicas, Serrano 121, 28006 Madrid, Spain; 2 GdS-Optronlab, Física de la Materia Condensada, Universidad de Valladolid, Paseo de Belén 19, 47011 Valladolid, Spain; 3 Departamento de Tecnología Electrónica, Universidad Carlos III de Madrid, Avda. de la Universidad 30, 28911 Leganés, Spain

**Keywords:** Radiation physics, Applied sciences, Devices

## Abstract

We present a novel class of narrow-band, polarization-selective terahertz (THz) absorbers and polarizers based on cloaked quasi-bound states in the continuum (qBICs) supported by doped germanium metasurfaces consisting of tilted microdisk and micropillar arrays that break out-of-plane symmetry, enabling impedance-matched conditions at specific Brewster angles. This configuration yields highly asymmetric absorption: near-complete transparency at one incidence angle and strong coupling to cloaked qBICs at the opposite angle. Microdisk arrays in TE polarization exhibit absorbance approaching 100%, while micropillar arrays in TM polarization reach ∼55% under similar conditions. The metasurfaces also work as efficient polarization filters, suppressing one polarization while transmitting the orthogonal one with near 100% efficiency. Additionally, we discuss practical implementation via obliquely grown Ge disks, along with strategies for active tuning of the asymmetry angle. These findings establish cloaked qBIC-based metasurfaces as a versatile platform for high-Q THz absorption, polarization control, and advanced sensing applications.

## Highlights

• THz metasurfaces made of doped-Ge inclined microcylinders that exploit impedance-matched, cloaked quasi-BICs
• Narrow-band, high-Q THz absorption with strong angular asymmetry
• Efficient polarization-selective filtering

## Graphical abstract

**Figure:**
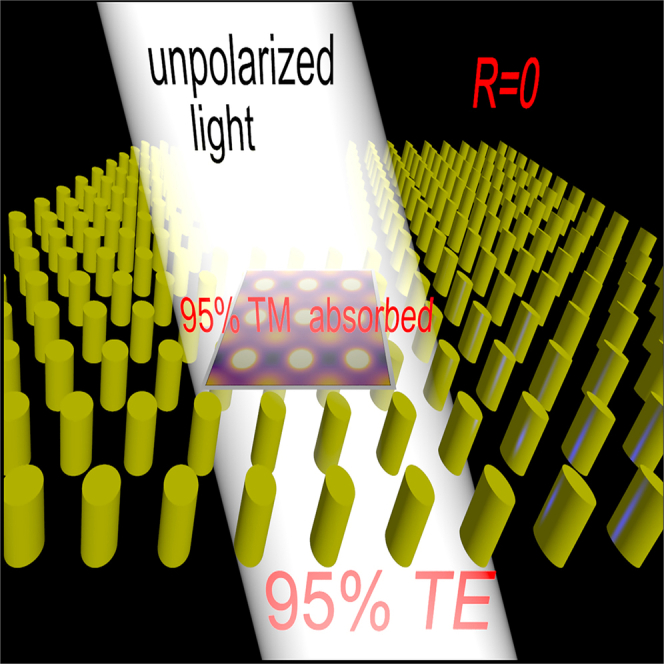

## Introduction

Metasurfaces have emerged as a state-of-the-art solution for planar terahertz (THz) absorbers due to their exceptional ability to manipulate electromagnetic waves at subwavelength scales.^1^^,^^2^ These engineered surfaces, composed of periodic or quasi-periodic arrays of resonant elements, enable precise control over absorption characteristics by tailoring the geometry, material composition, and arrangement of their unit cells. Recent advances have demonstrated ultra-thin, broadband, and polarization-insensitive THz absorbers with high efficiency, exploiting mechanisms such as impedance matching and resonance coupling.^3^^,^^4^^,^^5^^,^^6^ By contrast, narrow-band anisotropic metasurface absorbers represent a sub-field of THz absorption, primarily focused on sensing, filtering, and polarization-selective devices. This can be achieved by engineering a high Q factor resonance, often through the excitation of quasi-bound states in the continuum (qBICs).^7^^,^^8^^,^^9^^,^^10^^,^^11^^,^^12^^,^^13^^,^^14^^,^^15^^,^^16^^,^^17^ While high-Q factors lead to an extremely sharp absorption peak, which is the key enabler for ultra-sensitive applications, the inherent polarization-selective nature makes these structures ideal as perfect polarizers and polarization-sensitive modulators in integrated THz systems. Typically, narrow-band THz absorbers utilize complex structures with inherent geometric asymmetry^18^^,^^19^^,^^20^^,^^21^ such as stripes, elliptical shapes, L-shaped elements, or non-symmetrical apertures; therefore, attention should turn to simpler structures with similar capabilities.

Recently, the emergence of peculiar qBICs without impedance mismatch, called Brewster-like effect, has been reported in optical metasurfaces, especially with broken out-of-plane symmetry.^22^^,^^23^^,^^24^ These are based on single dielectric disk metasurfaces supporting dipolar vertical electric/magnetic BICs.^25^^,^^26^^,^^27^ By tilting meta-atoms (pillars or disks) in the plane of incidence, the qBIC band becomes reflectionless (impedance-matched) at fixed symmetric angles, but with an asymmetric behavior: it is fully transparent (uncoupled qBIC) at one angle, and strongly couples to the qBIC at the opposite one, which becomes effectively cloaked.^24^ At the same time, anisotropy results in a dramatically different absorption response for two orthogonal polarizations (transverse electric, TE, and transverse magnetic, TM). This allows the device to act as a perfect absorber for one polarization while being transparent or highly reflective for the other.

Here, we will show how to exploit such asymmetric, impedance-matched qBICs supported by doped Ge microdisk/micropillar arrays to induce high-Q fully asymmetric absorption. The proof-of-principle will be presented in Sec. II through calculations within the dipolar approximation for both polarizations, revealing the crucial role played by the impedance matching and the asymmetric qBIC excitation in the resulting absorption for tilted-cylinder arrays made of weakly doped germanium. Then, full numerical simulations will confirm the narrowband asymmetric behavior when replacing merely tilted meta-atoms by realistic inclined (obliquely grown) disk/pillars. Finally, means to actively switch and tune the asymmetry angle will be proposed.

## Results

### Impedance-matched cloaked THz qBICs

**Figure 1:**
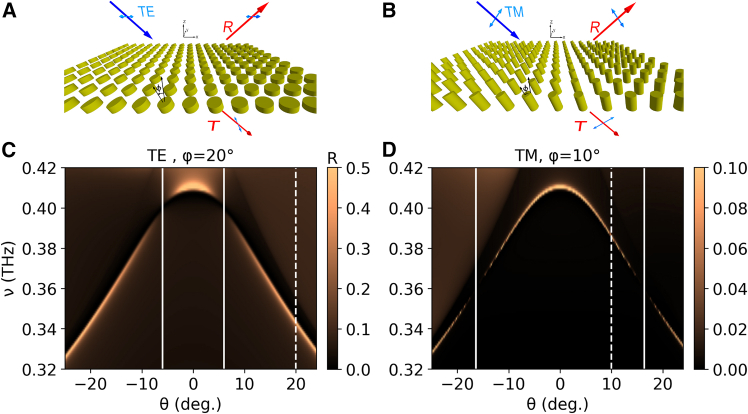
Schematics of the proposed THz metasurfaces and impedance matching principle for the lossless scenario (A and B) Schematics of the square arrays (period = 300 μm) studied henceforth, made of: (A) Ge tilted microdisks with height *h* = 50 μm and diameter *D* = 200 μm, and *φ* = 20°; and (B) Ge tilted micropillars with height *h* = 200 μm and diameter *D* = 100 μm, and *φ* = 10°. Color maps of the reflectance calculated using SMUTHI including up to the *l* = 2 (quadrupolar) contributions for square arrays (period *a* = 300 μm) of Ge tilted cylinders (*n* = 4.16) embedded in a uniform medium (*n* = 2.1): (C) microdisks with *h* = 50 μm and diameter *D* = 200 μm, and *φ* = 20° (TE polarization), and (D) micropillars with *h* = 200 μm and diameter *D* = 100 μm, and *φ* = 10° (TM polarization). Solid lines denote the effective Brewster angles *θ* = ±*φB* at which impedance matching occurs, the qBIC band thus becoming dark. Dashed lines indicate disk/pillar tilt angles, so that the corresponding incident plane waves are parallel to them.

By exploiting the physics underlying the emergence of cloaked qBICs with broken out-of-plane symmetry,^24^ and extrapolating dimensions to the desired THz regime, we focus on metasurfaces consisting of tilted microdisk or micropillar arrays to examine the mechanisms that lead to the formation of cloaked qBICs in both linear polarizations. A single germanium microcylinder is considered per unit cell, with two different geometrical shapes (see the schematics in Figures 1A and 1B): microdisks with height *h* = 50 μm and diameter *D* = 200 μm, or micropillars with height *h* = 200 μm and diameter *D* = 100 μm. To compose the array, the unit cells are arranged considering a square lattice with a period *a* = 300 μm. Throughout this work, we use the refractive index for crystalline Ge, *n* ≃ 4.1, assuming either a lossless response for undoped Ge or a small imaginary part, *k* = 0.02–0.05, depending on the (weak) level of doping (e.g., Sb).^28^ The arrays are embedded in a uniform medium with refractive index *n* = 2.1, which resembles a quartz substrate coated with a thick index matching layer.^29^

We use SMUTHI^30^ (scattering by multiple particles in thin-film systems), a free software based on the T-matrix method to account for the single particle scattering and on the scattering-matrix method for the propagation through a layered medium, to calculate the reflection (R) and transmission (T) coefficients as functions of THz frequency and angle of incidence. The frequency domain in all cases is in the non-diffractive regime. This implies that the wavevector-frequency regime is such that no diffraction orders are allowed other than the 0th order. Thus, *R* and *T* correspond to the specularly reflected and transmitted intensities, respectively, normalized by that of the incident beam. The results are shown in Figures 1C and 1D for the two square arrays (*a* = 300 μm) of undoped, lossless Ge microdisks and micropillars for TE and TM polarization, respectively. Only *R* is shown since, due to energy conservation in the absence of losses, *R* + *T* = 1. In both cases, the two lowest-order qBIC bands are observed, stemming from the lowest-order magnetic-dipole resonance for the microdisk array in TE polarization and the lowest-order electric-dipole resonance for the micropillar array in TM polarization, respectively. Moreover, due to out-of-plane symmetry breaking, transparent (dark) qBIC regions emerge near the so-called effective Brewster angles *θ* = ±*φ*_*B*_, denoted by solid vertical lines, which lie at *φ*_*B*_ = −6° for the microdisk array in TE polarization, and at *φ*_*B*_ = 16.4° for the micropillar array in TM polarization.

Note that such angles may differ from pure meta-atom tilt angles. If the vertical dipole resonance (oriented along the cylinder axis) responsible for the qBIC band is spectrally well isolated from the in-plane dipolar resonances, as demonstrated in the microwave regime for high refractive index (ceramic) disks, the resulting Brewster angle is identical to the tilt angle.^23^ However, when the in-plane and out-of-plane dipolar resonances lie spectrally close, as is the case of semiconductor cylinders from the visible to the low frequency regimes, lattice-induced interactions couple these modes such that the resulting qBIC band resembles an array of resonant dipoles with an effective dipole moment rotated with respect to the cylinder axis.^24^ Thereby, the corresponding effective Brewster angle becomes in this case that of the effective dipole moment orientation, so that *φ*_*B*_ ≠ *φ*. To further illustrate the behavior of the effective Brewster angle, we include in the supplemental information (see Figure S1) additional calculations of the reflectance analogous to those in Figures 1C and 1D, but for two different tilt angles. The first corresponds to the untilted case *φ* = 0° (Figures S1A and S1B), which reveals the emergence of the expected symmetry-protected BIC at the Γ point. The second corresponds to half of the tilt angles considered in Figure 1: *φ* = 10° for the microdisk array in Figure S1C and *φ* = 5° for the microdisk array in Figure S1D. Although no simple analytical expression exists to directly correlate these angles,^24^ an intuitive trend can be identified. For the micropillar array, the in-plane dipolar contributions are relatively weak, so that the effective Brewster angle remains close, though smaller, than the tilt angle. In contrast, for the microdisk array, the in-plane dipolar contributions are much stronger and induce large rotations that compensate for the geometric tilt, leading to effective Brewster angles that are closer to normal incidence.

Therefore, both planar arrays satisfy the impedance matching condition at *θ* = ±*φ*_*B*_. This condition is readily apparent from the definition of normalized impedance as *Z*/*Z*_0_ = (1 + *r*)/(1 − *r*), where *Z*_0_ is the impedance of the incident medium and *r* the complex reflection coefficient (*R* = |*r*|^2^). It follows that the impedance matching condition, *Z*/*Z*_0_ = 1, is satisfied when the reflection coefficient vanishes (*r* = 0), which is the case at the Brewster angles *θ* = ±*φ*_*B*_. Recall that the impedance matching condition at *θ* = ±*φ*_*B*_ in the absence of losses does not allow to distinguish between the excitation of a cloaked qBIC at *θ* = −*φ*_*B*_ and the fully uncoupled qBIC at *θ* = *φ*_*B*_, both revealed as a full transparency condition. This will become evident below upon introducing absorptive losses. The term *cloaked* qBIC was coined in Hidalgo-Arteaga et al.^24^ to describe a qBIC in a metasurface that, due to symmetry constraints, cannot radiate in the upper plane along the direction determined by its in-plane wavevector, the only allowed direction imposed by the array symmetry. The simplest example is a resonant mode supported by an array of dipoles with dipole moments oriented along such a specific direction,^23^ since dipoles do not radiate along their dipole axis. However, this mode does not constitute a true BIC, as it can still radiate into the lower half-space along the direction determined by its in-plane wavevector, which is the mirror image of that in the upper half-space. As a result, THz waves incident from the upper half-space can couple to this qBIC, but the mode cannot radiate in the specular reflection direction, leading to zero reflectance (*R* = 0). Importantly, radiation into the specular transmission direction remains allowed, and in the absence of losses, energy conservation requires full transmission (*T* = 1). Consequently, although the qBIC is excited, the metasurface remains fully transparent, in such a way that the qBIC can be considered *cloaked*.^24^

### Absorption

Let us now consider weakly lossy Ge meta-atoms with *n* = 4.16 + *ik*, with *k* = 0.025–0.05; these values are similar to those reported for Sb-doped germanium in the THz regime.^28^ We use different doping levels depending on the metasurface to achieve a similar degree of absorption: *k* = 0.025 for microdisks and *k* = 0.05 for micropillars. Even though their respective volumes are identical, lower/higher absorption is assumed for the qBIC resonance having larger/smaller concentration of electric field inside the metadisk/metapillar due to its MD/ED nature. The choice aims to optimize the performance as a compromise between absorption needed and resonance broadening. Nonetheless, a wide range of absorptive losses can be achieved by tuning the Sb-doping level of germanium.^28^

**Figure 2:**
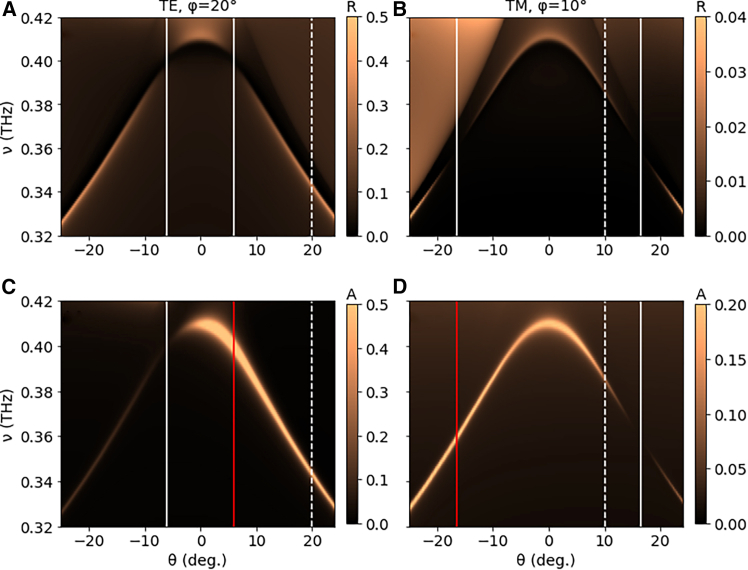
Reflectance and absorbance of the tilted lossy Ge disk metasurface Color maps of the reflectance (A and B) and absorbance (C and D) calculated using SMUTHI (up to the *l* = 2 quadrupolar contribution) for the two qBIC bands emerging in the two metasurfaces studied above in Figure 1, assuming now that the disks are made of doped (weakly lossy) Ge (*n* = 4.16 + *ik*), embedded in a uniform medium (*n* = 2.1): (A and C) *h* = 50 μm and diameter *D* = 200 μm, and *φ* = 20° (TE polarization), with *k* = 0.025; and (B and D) *h* = 200 μm and diameter *D* = 100 μm, and *φ* = 10° (TM polarization), with *k* = 0.05. Solid lines in (A)–(D) denote the effective Brewster angles *θ* = ±*φB* at which impedance matching occurs, the qBIC band thus becoming dark in reflection (A and B); red lines in (C and D) mark the angle of cloaked qBICs (large absorption), whereas the uncoupled qBICs (negligible absorption) remain in white. Dashed lines indicate disk/pillar tilt angles, so that the corresponding incident plane waves are parallel to them.

The results are shown in Figure 2 for weakly absorbing Ge meta-atoms with *k* = 0.025. Due to the presence of losses, both reflectance *R* (Figures 2A and 2B) and absorbance *A* = 1 − *R* − *T* (Figures 2C and 2D) are presented now. The qBIC bands are preserved in all cases for the corresponding polarizations, Figures 2A and 2C for the microdisk array in TE polarization and Figures 2B and 2D for the micropillar array in TM polarization. By examining the reflectance maps in Figures 2A and 2B, the qBIC bands appear weaker than those in the absence of losses (note the colormap scale), yet satisfying the angular symmetry and with the impedance-matching condition at *θ* = ±*φ*_*B*_ fully preserved (*R* = 0). By contrast, Figures 2C and 2D are clearly asymmetric, manifesting the strong asymmetric impact of absorption. The excitation of cloaked qBICs at *θ* = −*φ*_*B*_ (*φ*_*B*_ = 6° in Figure 2C and *φ*_*B*_ = −16.4° in Figure 2D, marked as red vertical lines, for the microdisk/micropillar array in TE/TM polarization, respectively) leads to remarkable absorptive losses, whereas at *θ* = *φ*_*B*_ (white vertical lines) the metasurfaces remain fully transparent (*T* = 1 − *R* − *A* = 1), since coupling into the qBIC is forbidden.

**Figure 3:**
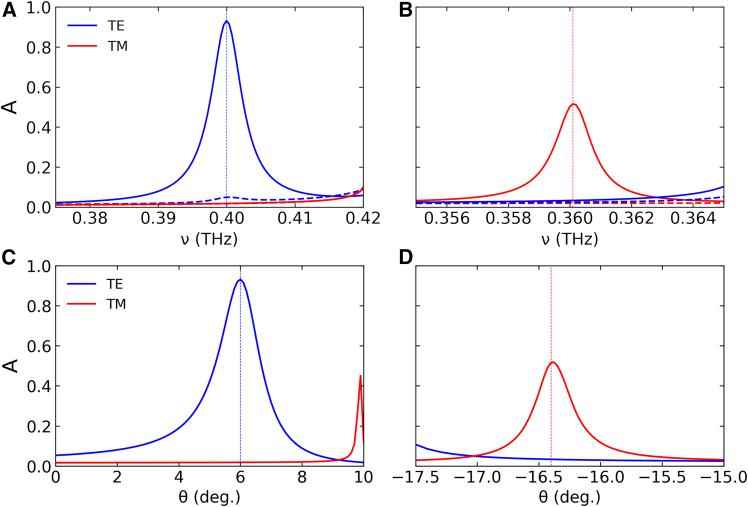
Polarization dependence of the absorbance for the cloaking and transparency conditions (A and B) Absorbance spectra in both polarizations at fixed angles of incidence, *θ* = ±*φB*, including the cloaking (solid curves) and transparency (dashed curves) conditions for: (A) microdisk array at *θ* = ±6° and (B) micropillar array at ±*θ* = 16.4°. Vertical dotted lines indicate the Brewster angle frequencies *ν* = *νB*. (C and D) Absorbance in both polarizations as a function of the angle of incidence (close to the cloaked qBIC condition, *θ* = −*φB*) for fixed Brewster angle frequencies: (C) *ν* ∼ 0.4 THz for the microdisk array and (D) *ν* ∼ 0.36 THz for the micropillar array. Vertical dotted lines indicate the cloaked qBIC angles *θ* = −*φB* Arrays made of doped (weakly lossy) Ge (*n* = 4.16 + *ik*) cylinders, embedded in a uniform medium (*n* = 2.1): (A and B) *h* = 50 μm, *D* = 200 μm, and *φ* = 20°, with *k* = 0.025; and (C and D) *h* = 200 μm, *D* = 100 μm, and *φ* = 10°, with *k* = 0.05. In all cases, blue/red curves represent TE/TM polarizations, respectively.

Let us examine in detail the absorption at Brewster angles. To this end, we plot the spectral dependence of *R* and *T* at the incidence angles where the impedance-matching condition leads to the emergence of such asymmetric features in the qBIC bands. This is shown in Figures 3A and 3B, including both polarizations for each metasurface for the sake of completeness. The spectra at *θ* = −*φ*_*B*_, namely, *θ* = 6° for TE polarization in Figure 3A and *θ* = −16.4° for TM polarization in Figure 3B, show large, narrow absorption peaks precisely at the frequency of the corresponding qBIC bands, *ν* ∼ 0.4 THz with a Q factor of ∼20 in Figure 3A, and *ν* ∼ 0.36 THz with a Q factor of ∼500 in Figure 3B. By contrast, the absorbance spectra for the symmetric angles, *θ* = −6° for TE polarization in Figure 3A and *θ* = 16.4° for TM polarization in Figure 3B, show negligible absorption as a result of the forbidden coupling into the qBIC, clearly demonstrating the strong asymmetry in absorption. To shed some light on this asymmetric absorption, we next plot in Figures 3C and 3D the absorbance versus the angle of incidence for the frequencies at which the Brewster angles emerge: *ν* ∼ 0.4 THz in Figure 3C and *ν* ∼ 0.36 THz in Figure 3D. Again, large absorbance peaks emerge at the cloaked qBIC angles in the polarization corresponding to the qBIC bands of each metasurface: *θ* = −*φ*_*B*_ = 6° for TE polarization in Figure 3C and *θ* = −*φ*_*B*_ = −16.4° for TM polarization in Figure 3D, being nearly lossless at the symmetric angles. Incidentally, surface lattice resonances^31^ produce some nearly symmetric absorption peaks in the complementary linear polarizations at angles close to the diffraction lines, observed in Figure 3C for TM polarization at *θ* ≈ 10° and in Figure 3D for TE polarization at *θ* ≤ −17.5°.

Interestingly, note that absorbances close to 100% are observed for the microdisk array in TE polarization. This largely exceeds the fundamental limit of 50% stated for resonances stemming from single dipolar contributions at normal incidence.^32^ Apart from the fact that the incident angle is slightly off-normal, the impedance-matching condition that occurs at cloaked qBICs due to the out-of-plane symmetry breaking allows for such nearly total absorption, blocking the upper radiation channels. Also, note that larger Ge doping would be needed to increase the absorbance of the micropillar array in TM polarization, because the ED resonance originating from the qBIC band is more delocalized outside the Ge micropillar, as compared to the highly localized MD resonance within Ge microdisks.

### Polarization filtering

**Figure 4:**
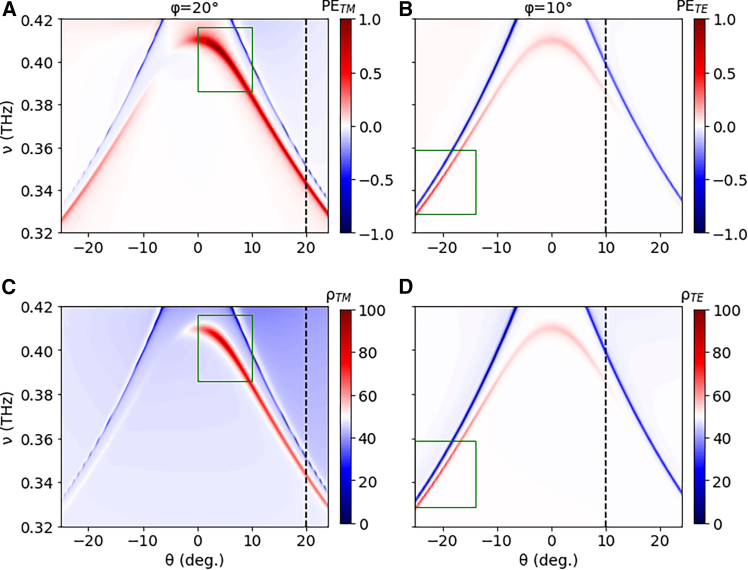
Polarizer performance of the tilted lossy Ge disk metasurface Color maps of the polarizer performance (A and B) and transmission rate (C and D) calculated using SMUTHI (up to the l = 2 quadrupolar contribution) for the two qBIC bands emerging in the metasurfaces studied above in Figure 2, made of doped (weakly lossy) Ge (*n* = 4.16 + *ik*) tilted micro-cylinders, embedded in a uniform medium (*n* = 2.1). (A and C) microdisks with *h* = 50 μm and diameter *D* = 200 μm, and *φ* = 20° (TM-pass filtering), with *k* = 0.025; micropillars with and (B and D) *h* = 200 μm and diameter *D* = 100 μm, and *φ* = 10° (TE-pass filtering), with *k* = 0.05. Green boxes mark the regions close to the angle of cloaked qBICs (large absorption), *θ* = ±*φB*. Dashed lines indicate disk/pillar tilt angles, so that the corresponding incident plane waves are parallel to them.

We now explore the performance of the above metasurfaces as polarization filters assuming unpolarized incident THz radiation. To quantify it, we define two magnitudes, *PE* and *ρ*. The former (polarization efficiency, *PE*) quantifies the normalized transmission contrast between two orthogonal linear polarizations, while the latter (*ρ*) represents the percentage of the total non-absorbed energy transmitted in the selected polarization state, as:

(Equation 1) $$\begin{array}{ccc} & & PE_{\alpha }=\frac{T_{\alpha }-T_{\beta }}{T_{\alpha }+T_{\beta }}, \end{array}$$

(Equation 2) $$\begin{array}{ccc} & & \rho_{\alpha }=\frac{T_{\alpha }}{R_{\alpha }+R_{\beta }+T_{\alpha }+T_{\beta }}*100, \end{array}$$

where *α*, *β* = TE, TM stand for each linear polarization; note that *PE*_TE_ = −*PE*_TM_. Both quantities are plotted as color maps in Figure 4 for the arrays of microdisks (Figures 4A and 4C) and micropillars (Figures 4C and 4D), previously studied in Figure 2. In both cases, we observe two bands arising from the qBIC bands of interest and the SLRs near the diffraction limits, within a background of nearly unpolarized, lossless transmission (*PE* = 0, *ρ*_*α*_ = 50%). First, let us focus on the rectangular green boxes denoting the *ν*, *θ* regions where the cloaked qBICs with large absorption are located. Performances approaching unity in the desired polarization (opposite to that of the corresponding qBIC band) are observed therein, in TM polarization for the microdisk array in Figure 4A and in TE polarization for the micropillar array in Figure 4B. The cloaked qBIC supported by the microdisk array absorbs about 95% of incident TE polarization (see Figures 3A and 3C), leading to *PE*_TM_ ∼ 0.95 in Figure 4A; in the case of the cloaked qBIC of the micropillar array, about 50% of incident TM polarization is absorbed (see Figures 3B and 3D), leading to *PE*_TE_ ∼ 0.5 in Figure 4B. Moreover, Figures 4C and 4D show in turn that such linearly polarized transmission carries nearly all the radiated energy, due to the fact that the reflectance is negligible in both polarizations at the cloaked qBIC bands, resulting in a highly efficient polarization. In addition, if we look at the mirror rotated angles, the condition of full transparency leads to unpolarized light being transmitted without interacting with the metasurfaces. Incidentally, note that impedance matching also makes the efficiency higher around the cloaked qBIC, being significantly smaller in other regions of the qBIC band where this condition is not fulfilled. The qBIC-induced reflection thus allows for an unwanted radiation channel where performance deteriorates, being of the order of 50% at most. In addition, note that SLRs can also perform as polarization filters, but behave similarly to normal qBICs, far from the cloaked qBIC condition, thus limiting performance in transmission (<40%) due to reflection losses. In those regions, both normal qBICs and SLRs can play the role of narrow-band polarization filters in reflection, at the expense of limited performance <50%.

### Implementation: obliquely grown disk metasurfaces

In practical implementations, the idealized tilted cylindrical meta-atoms considered in the dipolar model must be replaced by inclined microdisks/micropillars whose axes are physically rotated during fabrication. Obliquely grown semiconductor microstructures fabricated, for example, through tilted reactive ion etching, gray-scale lithography, or lasing ablation,^33^^,^^34^^,^^35^^,^^36^^,^^37^ naturally produce such inclined geometries (with moderate aspect ratios) and thereby preserve the essential out-of-plane symmetry breaking that enables cloaked quasi-BIC formation.

**Figure 5:**
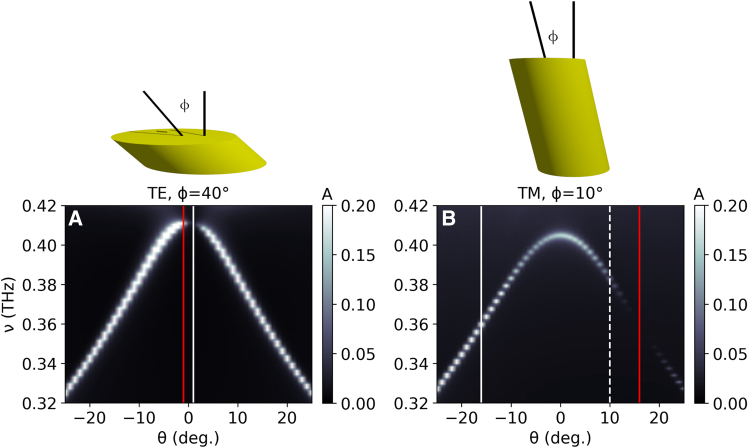
Absorbance of the inclined lossy Ge disk/pillar metasurface Top: geometry of the inclined disks (left) and pillars (right). (A and B) Color maps of the absorbance calculated using FDTD for the qBIC bands emerging in two metasurfaces made of doped (weakly lossy) Ge (*n* = 4.16 + *ik*) inclined (rather than tilted) micro-disks/-pillars, embedded in a uniform medium (*n* = 2.1): (A) *h* = 50 μm and diameter *D* = 200 μm, and *ϕ* = 40° (TE polarization), with *k* = 0.025; and (B) *h* = 200 μm and diameter *D* = 100 μm, and *ϕ* = 10° (TM polarization), with *k* = 0.05. Solid lines denote the effective Brewster angles *θ* = ±*ϕB* at which impedance matching occurs: those in red mark the angle of cloaked qBICs (large absorption), whereas those in white mark the angle of the uncoupled qBICs (negligible absorption). Dashed lines indicate disk/pillar inclination angles *ϕ*, so that the corresponding incident plane waves are parallel to them: the one for inclined disks lies outside the corresponding angular range shown in (A).

Full-wave FDTD simulations of these realistic inclined elements confirm that the key mechanism survives: the qBIC band remains threaded by two effective Brewster angles, *θ* = ±*ϕ*_*B*_ (note that we use *ϕ*_*B*_ for inclined cylinder metasurfaces, rather than *φ*_*B*_ used above for tilted ones), at which the metasurface becomes impedance-matched to its environment. As in the tilted case, these angles are not strictly equal to the geometric inclination angle *ϕ*, due to contributions from in-plane electric and magnetic dipoles; nevertheless, they accurately mark the transition between uncoupled and strongly coupled qBIC regimes. This is shown in Figure 5 for arrays of inclined microdisks and micropillars (*ϕ* = 40° and *ϕ* = 10°, respectively), where only the absorbance is represented. When the incident wave approaches the dark Brewster angle (*ϕ*_*B*_), the reflection channel vanishes and the inclined disks/pillars become fully transparent, absorption being negligible (uncoupled qBIC). This becomes evident in *θ* = *ϕ*_*B*_ = 1° for TE polarization in Figure 5A and *θ* = *ϕ*_*B*_ = 16° for TM polarization in Figure 5B. At the opposite Brewster angle (−*ϕ*_*B*_), by contrast, impedance matching funnels energy directly into the cloaked qBIC, producing a sharp enhanced absorption peak whose amplitude and spectral width agree with those obtained from the analytical tilted-dipole description. Nonetheless, whereas the Brewster angle for inclined micropillars is nearly identical to that for tilted micropillars (*ϕ*_*B*_ ∼ *φ*_*B*_, see Figures 2D and 5B, this is not the case when comparing inclined microdisks; Figure 2C, with tilted microdisks (see Figure 5A). This is due to the fact that inclined planar cylinders do not effectively tilt the MD resonance (stemming from electric field circulation within the disk), as tilted microdisks do, even if the inclination angle is much larger (*ϕ* = 2*φ* = 20°); by contrast, inclined micropillars with *ϕ* = *φ* = 1° tilt the ED resonance as efficiently as tilted micropillars do.^24^ Therefore, FDTD calculations for micropillar-based TM qBICs show this strong angular asymmetry as prominently as in the idealized models, demonstrating that oblique growth does not degrade, but rather robustly preserves the underlying Brewster-mediated cloaking physics. Finally, planar microdisks have to be inclined much more than tilted ones in order to achieve similar impedance-matched TE qBICs.

**Figure 6:**
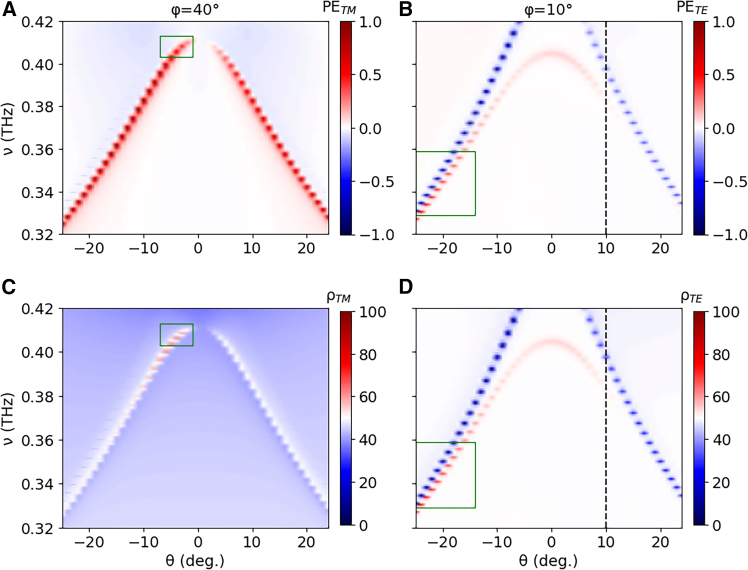
Polarizer performance of the inclined lossy Ge disk metasurface Color maps of the polarizer performance (A and B) and transmission rate (C and D) calculated using FDTD for the qBIC band emerging in the metasurfaces studied above in Figure 5, made of doped (weakly lossy) Ge (*n* = 4.16 + i0.05) inclined disks/pillars, embedded in a uniform medium (*n* = 2.1), for: (A and C) TM-pass filtering, microdisks with *h* = 50 μm and diameter *D* = 200 μm, and inclination angle *ϕ* = 40°; (B and D) TE-pass filtering, micropillars with *h* = 200 μm and diameter *D* = 100 μm, and inclination angle *ϕ* = 10°. Green boxes mark the regions close to the angle of cloaked qBICs (large absorption), *θ* = ±*ϕB*. Dashed lines indicate the pillar inclination angle *ϕ* = 10°, so that the corresponding incident plane waves are parallel to it: the ones for inclined disks lie outside the corresponding angular range shown in (A and C).

Beyond validating the absorption asymmetry, arrays of inclined disks and pillars also exhibit polarization-filter performance near the cloaked qBIC angles. Because the Brewster-matched condition strongly suppresses reflection for both polarizations, the metasurface behaves as a purely absorptive filter in the polarization corresponding to the qBIC band, while transmitting the orthogonal polarization with minimal loss. We include the polarization performance and transmission rate in Figure 6 for both inclined microdisks/micropillars. The MD-type qBIC drains TE-polarized energy, producing an efficient TM-pass filter near *θ* ≈ − *ϕ*_*B*_ = −1°, shown in Figures 6A and 6C; whereas the ED-type qBIC drains TM-polarized energy, producing an efficient TE-pass filter near *θ* ≈ − *ϕ*_*B*_ = −17°, shown in Figures 6B and 6D. Importantly, the inclined geometry maintains the asymmetric nature of the filtering effect: rotating to the opposite angle (*θ* ≈ *ϕ*_*B*_) switches coupling to the qBIC off, so the metasurface becomes nearly polarization-insensitive and fully transmitting. Note that the filtering performance of the micropillar-based TM qBICs is significantly better than that for microdisk-based TE qBIC, due to the fact that, as commented above, the former shows the strong angular asymmetry as prominently as in the idealized models with tilted microcylinders. In summary, this angular switching behavior is experimentally attractive, as it allows the same device to operate either as a highly selective narrow-band polarizer or as a transparent window simply by adjusting the incidence angle. Furthermore, because the geometry is defined solely by the inclination of the meta-atoms, the performance can be tuned by modifying the growth angle, enabling wafer-level fabrication of reconfigurable THz absorbers and polarization filters based on cloaked qBICs.

## Discussion

This study introduces a new class of terahertz (THz) metasurfaces that exploit cloaked qBICs to achieve narrow-band, angular- and polarization-selective absorption and filtering. The metasurfaces consist of arrays of tilted germanium microdisks and micropillars, which break out-of-plane symmetry to enable impedance-matched conditions at specific Brewster angles. This symmetry breaking produces a unique asymmetric response: at one incidence angle, the qBIC remains uncoupled, resulting in full transparency, while at the opposite angle, the cloaked qBIC is strongly excited, yielding high-Q absorption.

Using a combination of dipolar models and full-wave numerical simulations, it is demonstrated that weakly Sb-doped Ge meta-atoms (with refractive index *n* = 4.16 + *ik*, *k* = 0.025–0.05) support sharp absorption peaks at frequencies around 0.36–0.40 THz. Microdisk arrays in TE polarization achieve absorbance close to 100%, exceeding the theoretical 50% limit for single-dipole resonances due to impedance matching, while micropillar arrays in TM polarization reach ∼55%, with Q factors above 10^2^. The metasurfaces also act as efficient asymmetric narrow-band polarization filters: microdisk/micropillar arrays filter TM/TE polarization at the incident angle and frequency of the cloaked qBIC, being however fully transparent at the opposite angle. Thus, they transmit one linear polarization with near-unity efficiency while suppressing the orthogonal component, making them ideal for integrated THz systems.

From a fabrication standpoint, several established microstructuring techniques enable the controlled formation of inclined semiconductor microdisks and micropillars with moderate aspect ratios in the range of 100 μm, suitable for cloaked qBIC THz metasurfaces. It is worth noting that doped Si or other semiconductors with a similar refractive index can be used as alternatives to Ge. Tilted deep reactive ion etching (DRIE) is likely the most suitable for semiconductor-based implementations. Slanted profiles can be achieved by employing a tilted electrode or a Faraday cage (a metal mesh) placed over the wafer. The mesh bends the plasma electric field lines, forcing the ions to strike the wafer at a specific angle, thereby sculpting pre-patterned Ge mesas into slanted microdisks via directional etchprofiles,^33^^,^^34^ providing precise control over the height-to-diameter aspect ratio. Alternatively, ultrafast laser ablation represents a competitive approach in terms of cost and processing speed.^35^ The laser head or the stage can be tilted to achieve slant. Since the required period is quite large (∼400 μm) and the tilt angles moderate, there is plenty of clearance for the laser beam to hit the substrate at an angle without being blocked by neighboring cylinders. As a drawback, the performance tolerance to imperfections of the resulting metasurfaces should be analyzed, since the sidewalls may have some slight re-deposition (slag), reducing surface smoothness compared to etched structures. Importantly, these fabrication routes preserve the dielectric quality of weakly doped semiconductors, maintaining the low-loss conditions required for high-Q qBIC excitation. The resulting inclined arrays thus form a practical and scalable platform for implementing cloaked qBIC absorbers and polarization-selective THz components. Furthermore, alternative strategies based on electro- or magneto-optical tuning can be envisioned to dynamically control the effective asymmetry angle of otherwise untilted microstructures, enabling reconfigurable devices. A magneto-optical tuning mechanism in the visible domain has already been proposed.^38^ Such angular switching behavior is particularly appealing from an experimental standpoint, as it allows a single device to operate either as a highly selective narrowband polarizer or as a transparent window, simply by adjusting the angle of incidence or the external tuning mechanism.

Potential applications include ultra-sensitive THz sensing and spectroscopy, secure wireless communications, imaging and security screening, and dynamic polarization control. These findings position cloaked qBIC-based metasurfaces as a versatile platform for next-generation THz photonic technologies.

### Limitations of the study

From the theoretical analysis, notwithstanding the fact that the full numerical simulations confirm the performance, there are certain limitations for the inclined microdisk array for TM-pass filtering due to the limited range of available Brewster angles. In addition, the fabrication of uniform, large-area metasurfaces of inclined micro-cylinders with precise control of the inclination angle (as proposed in this study) is challenging; qBIC-based phenomenology, though, is quite robust due to its topological protection, so that a moderate number of unit cells suffices to manifest itself.

## Resource availability

### Lead contact

Requests for further information and resources should be directed to and will be fulfilled by the lead contact, José A. Sánchez-Gil (j.sanchez@csic.es).

### Materials availability

This study did not generate new materials.

### Data and code availability

• All data reported in this paper will be shared by the lead contact upon request.
• All original code is available from the lead contact upon reasonable request.
• Any additional information required to reanalyze the data reported in this paper is available from the lead contact upon request.

## Acknowledgments

A.B. thanks MICINN for the Ramón y Cajal Fellowship (grant no. RYC2021-030880-I). Financial support is acknowledged from the grants TED2021-130786B-I00, PID2022-137857NA-100, and PID2022-137569NB-C41 (LIGHTCOMPAS), funded by MCIN/AEI/10.13039/501100011033, “ERDF A way of making Europe,” and European Union NextGenerationEU/PRTR.

## Author contributions

Conceptualization, J.A.S.; methodology, J.L.P., A.B., and J.A.S.; investigation, L.H., J.L.P., B.G., A.B., S.C.P., and J.A.S.; writing – original draft, J.A.S.; writing – review and editing, J.L.P., B.G., A.B., and J.A.S.; funding acquisition, B.G., A.B., S.C.P., and J.A.S.; resources, A.B. and J.A.S.; supervision, J.A.S.

## Declaration of interests

The authors declare no competing interests.

## Declaration of generative AI and AI-assisted technologies in the writing process

During the preparation of this work, the authors used Copilot in order to explore the literature of THz absorbers and polish the English grammar in the “introduction.” After using this tool or service, the authors reviewed and edited the content as needed and take full responsibility for the content of the publication.

## STAR★Methods

### Key resources table

**Table:** 

REAGENT or RESOURCE	SOURCE	IDENTIFIER
**Software and algorithms**		

Python version 3.10	Python Software Foundation	https://www.python.org
SMUTHI (scattering by multiple particles in thin-film systems)	Egel et al.^30^	https://smuthi.readthedocs.io/
Lumerical FDTD	Ansys	https://www.ansys.com/products/optics/fdtd

### Method details

#### SMUTHI calculations

SMUTHI (scattering by multiple particles in thin-film systems) is a free software based on the T-matrix method to account for the single particle scattering and on the scattering-matrix method for the propagation through a layered medium.^30^ In particular, we made use of the modules calculating the power reflected and transmitted through an infinite planar array of cylindrical scatterers.

#### FDTD calculations

The full numerical simulations for realistic (inclined disk/pillar) metasurfaces have been carried out through the commercial software Lumerical (with institutional license), which provides a rigorous, full vector solution of Maxwell’s equations using the Finite-Difference Time-Domain method.

The transmission and reflection spectra were evaluated by simulating a single unit cell of the metasurface with periodic (Bloch) boundary conditions applied along the *x* and *y* directions, accounting for the in-plane wavevector. Perfectly matched layers (PMLs) were used along the out-of-plane (*z*) direction to suppress spurious reflections.

Oblique incidence was implemented using a broadband fixed-angle source technique (BFAST), which introduces the appropriate phase shift between adjacent unit cells. The structure was illuminated from the top by a plane wave incident at an angle *θ* with respect to the surface normal.

The transmitted and reflected power were recorded using monitors placed below the particle and above the source region, respectively.

### Quantification and statistical analysis

Our study does not include any statistical analysis.
