# Supplementary material for: Narrow-band asymmetric THz absorbers and polarizers based on impedance-matched, cloaked quasi-BICs in doped Ge microdisk metasurfaces
Source: iScience. 2026 Jun 5;29(6):116169. doi: 10.1016/j.isci.2026.116169 (PMC13253186; doi:10.1016/j.isci.2026.116169)
Supplement: Document S1. Figure S1 [file mmc1.pdf]

iScience, Volume *29*

Supplemental information

Narrow-band asymmetric THz absorbers and polarizers based on impedance-matched, cloaked quasi-BICs in doped Ge microdisk metasurfaces

Lucía Hidalgo-Arteaga, Jose L. Pura, Braulio García-Cámara, Ángela Barreda, and José A. Sánchez-Gil

SUPPLEMENTAL FIGURE S1

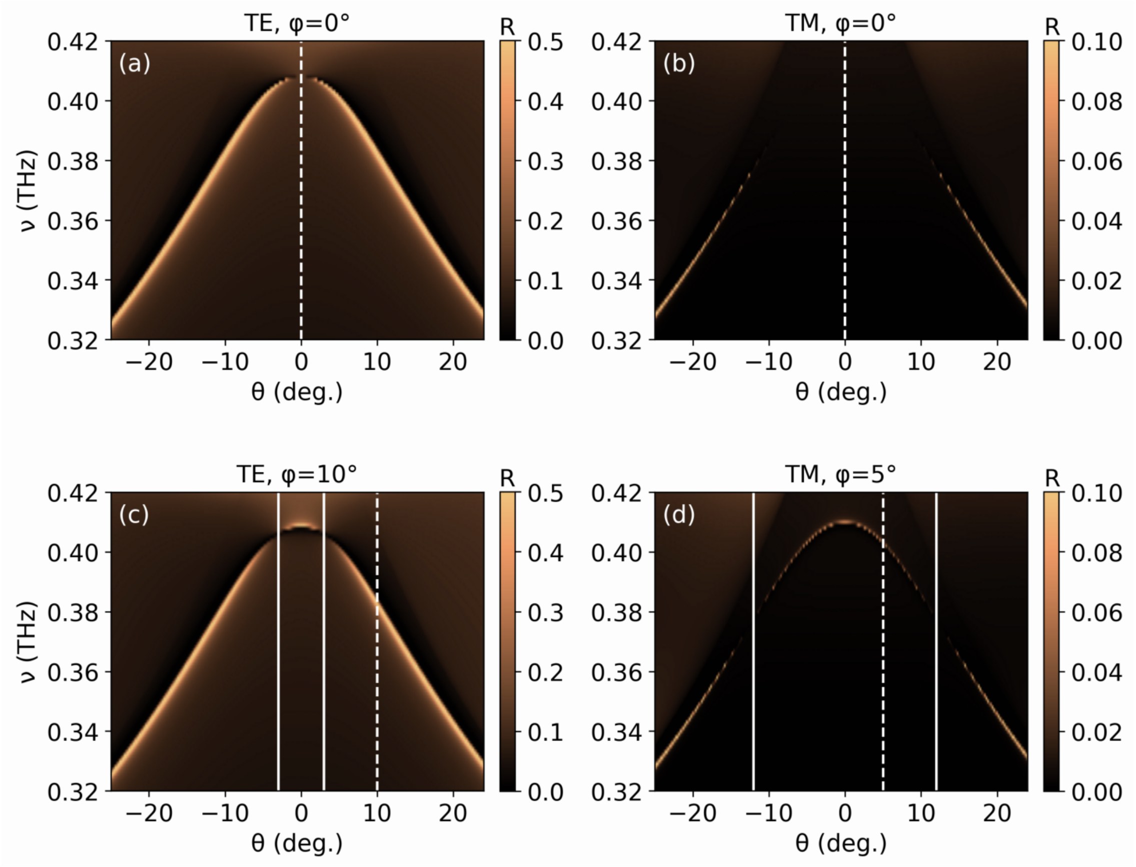

Reflectance of the lossless Ge disk metasurface revealing the impedance matching principle for two different tilt angles.

Color maps of the reflectance for Ge lossless disk/pillar metasurfaces as in Figure 1C (a,c) and Figure 1D (b,d), but for two different tilt angles: (a,b) same as Figure 1C (TE polarization) and Figure 1D (TM polarization), respectively, both for φ = 0º (untilted); (c) same as Figure 1C (TE polarization) for φ = 10º; same as Figure 1D (TM polarization) for φ = 5º.
